# Body Image Disturbance in Acromegaly Patients Compared to Nonfunctioning Pituitary Adenoma Patients and Controls

**DOI:** 10.1155/2015/624872

**Published:** 2015-05-20

**Authors:** Helen M. Conaglen, Dennis de Jong, Veronica Crawford, Marianne S. Elston, John V. Conaglen

**Affiliations:** ^1^Waikato Clinical School, University of Auckland, Private Bag 3200, Hamilton 3240, New Zealand; ^2^University of Waikato, Private Bag 3105, Hamilton 3240, New Zealand; ^3^Waikato Hospital, Private Bag 3200, Hamilton 3240, New Zealand

## Abstract

*Purpose*. Excess growth hormone secretion in adults results in acromegaly, a condition in which multiple physical changes occur including bony and soft tissue overgrowth. Over time these changes can markedly alter a person's appearance. The aim of this study was to compare body image disturbance in patients with acromegaly to those with nonfunctioning pituitary adenomas (NFAs) and controls and assess the impact of obesity in these groups. *Methods*. A cross-sectional survey including quality of life, body image disturbance, anxiety and depression measures, growth hormone, and BMI measurement was carried out. *Results*. The groups did not differ with respect to body image disturbance. However separate analysis of obese participants demonstrated relationships between mood scales, body image disturbance, and pain issues, particularly for acromegaly patients. *Conclusions*. While the primary hypothesis that acromegaly might be associated with body image disturbance was not borne out, we have shown that obesity together with acromegaly and NFA can be associated with body image issues, suggesting that BMI rather than primary diagnosis might better indicate whether patients might experience body image disturbance problems.

## 1. Introduction

Acromegaly is an uncommon disease (estimated prevalence of 4–13 cases per 100,000 population) [1, 2] invariably due to a GH-secreting pituitary adenoma. Excess GH production results in alterations in a person's physical appearance, including enlargement of facial features (nose, lips, ears, jaw, and forehead) as well as enlargement of the hands and feet [3]. Less visible symptoms of acromegaly include obstructive sleep apnea, excessive sweating, premature osteoarthritis, hypertension, cardiomegaly, diabetes, and an increased risk of benign and malignant tumors [4, 5]. Given the symptoms associated with acromegaly, it is unsurprising that the disease has been reported to have negative effects on both self-image and quality of life (QoL) [6, 7].

Despite the potential for acromegaly to affect a person's QoL in a number of ways, there is limited published research on this topic [8]. The development of a questionnaire specifically designed to measure the effects of acromegaly on QoL (AcroQoL) enabled dimensions of QoL important in acromegaly to be measured, demonstrating reduced QoL in those with acromegaly when compared to a normal population [9, 10]. The effects of disease control on QoL in acromegaly have proven to be mixed, with one published study showing an improvement in QoL after successful disease control [11], while another reported a reduction in QoL when radiotherapy was employed, even despite successful control of GH levels [12]. These equivocal findings may be due to a lack of sensitivity of the AcroQoL measure, the influence of comorbidities, or cosmetic and orthopedic deformities [11].

Perhaps due to the relative rarity of acromegaly, there is a paucity of literature regarding the effects of acromegaly on body image; one case report noted that body image was adversely affected in a person diagnosed with acromegaly [13]. A more recent study examined the impact of exercise on body image in 11 patients with acromegaly, finding a positive impact on self-assessed body perception after three months of exercise [14]. Further, it is reported that biochemically controlled acromegaly patients typically show higher measurements of obesity, specifically visceral adiposity, than age- and gender-matched controls [15].

Several other conditions that affect one's appearance have been reported to negatively impact body image (BI), for example, obesity [16, 17], psoriasis [18], systemic lupus erythematous [19], Cushing's syndrome [20], and rheumatoid arthritis [21]. In obese persons, identifying those who might respond to an intervention aimed at reducing body image disturbance or dissatisfaction is important when combining an operation with psychological interventions to assist the patient. Given that acromegaly also negatively affects a person's appearance, the condition might also lead to BI disturbance. If confirmed, then interventions aimed at helping patients to adjust their body image would potentially be beneficial when combined with medical control of acromegaly. However, to date, no studies have used the Body Image Disturbance Questionnaire (BIDQ) [22] investigating people with acromegaly compared to other groups.

GH deficits have been associated with reductions in QoL and psychological functioning, particularly anxiety and depression, while excess GH has been linked with changes in personality, mood lability, and depression [23]. Even when GH excesses or deficiencies are treated, psychopathology and QoL may remain problematic, although the literature is equivocal about this [24]. However a recent study of nonfunctioning pituitary adenomas (NFAs) found that QoL was similar to those without endocrine disease once the disorder is sufficiently treated [25]. These patients, in whom adenomas do not secrete excess GH, provide a useful comparison group for investigating the quality of life and body image disturbance effects of the two types of pituitary tumors on patients.

Existing literature suggests that patients with acromegaly have a greater impairment in QoL than those with NFAs and control participants, but that those with NFAs will not have QoL different to that of control participants [23]. The current study aimed to test the hypothesis that body image disturbance would be more likely in acromegaly patients than the NFA patients and controls, because of the disfiguring nature of acromegaly. The impact of obesity on body image disturbance in patients with both conditions, as well as the controls, is also examined.

## 2. Materials and Methods

### 2.1. Study Sample

Endocrine clinic medical records were used to identify a convenience sample of participants diagnosed with acromegaly or NFAs, who were invited to participate in the study face-to-face at routine clinic appointments, or by mail. Control participants were recruited among family and associates of the patient groups using a snowball sampling technique to ensure that participant groups were closely matched with respect to socioeconomic status and ethnicity.

### 2.2. Procedure

This was a cross-sectional survey of three groups, patients with acromegaly, patients with NFAs, and a control group. All participants signed an informed consent, and the study was conducted in accordance with the National Health Advisory Committee's Ethical Guidelines for Observational Studies and permission of the Endocrine Department. Questionnaires were administered over a period of approximately 6 months in 2012-13. Participants in all groups were mailed the questionnaire to complete at home, or they were given the questionnaire to complete while they were waiting to attend a clinic appointment. The majority of those invited participated in the study. Participants who agreed to a blood sample had these completed either prior to clinic appointments (patient groups), or following completion of the questionnaire (control group). GH and IGF1 results were used to confirm diagnoses or treatment progress, and in nonacromegaly participants, to ensure there was no evidence of excess GH production.

### 2.3. Instruments

The questionnaire set consisted of demographic information (including age, gender, ethnicity, height, weight, and relationship and employment status, as well as information about symptoms and comorbidities within the patient groups), together with the following measures:

1. A single item self-esteem query (“I have high self-esteem”) with a Likert response anchored by “Not at all true of me” and “Very true of me”; this was chosen because of the known connection between body image problems and low self-esteem [26–28].
2. The Hospital Anxiety and Depression Scale (HADS). This was developed to detect signs of depression and anxiety in outpatients, has been used widely in medical contexts, and is reputed to have good reliability and validity [29, 30].
3. The Body Image Disturbance Questionnaire (BIDQ) contains seven scaled items that pertain to appearance-related concerns, mental preoccupation with these concerns, associated experiences of emotional distress, resultant impairment in social, occupational, or other important areas of functioning, interference with social life or with school, job, or role functioning, and consequential behavioral avoidance [22]; the measure yields qualitative and quantitative responses and the mean scale scores (higher scores = more disturbance) allow comparison between the present study groups.
4. The Duke Health Profile (DHP) is a 17-item scale that contains six health subscales (physical, mental, social, general, perceived health, and self-esteem) and four dysfunction scales (anxiety, depression, pain, and disability) [31]. The scale's authors report that the mean score for patients with painful health issues was 58.1, compared to a mean of 83.9 for patients visiting their family practice for health maintenance. This general QoL measure allows within-study comparisons between groups and scores can also be compared with those from that reference sample of general outpatients.

### 2.4. Analyses

All completed questionnaires were scored and analyzed using Statistica version 11 (Statsoft Inc., Tulsa, OK, USA). One-way ANOVA was used to confirm the age-matched nature of the patient groups. The scores on all measures recorded by the three groups within the study were compared using nonparametric analyses as appropriate (Mann-Whitney* U* test or Kruskall-Wallis ANOVA by ranks). Within-group analyses examined acromegaly patients with respect to their IGF-1 status. Kruskall-Wallis analysis was used to examine between group differences among the obese participants within the study. A *p* value < .05 was considered to be statistically significant.

## 3. Results

### 3.1. Participants

A total of 82 participants took part in the study: 32 participants with diagnosed acromegaly, 29 participants with NFAs, and 21 control participants. The majority of the acromegaly and NFA patients had been diagnosed between 1977 and 2012 by the endocrine service; thus, time since onset varied across the sample. There was no significant difference between the mean ages of the two patient groups. The demographic characteristics of the patient and control groups are shown in Table 1.

### 3.2. Acromegaly Patients

From the patient symptom questionnaires the most frequently identified acromegaly features at diagnosis were enlarging of the hands and/or feet and visual acuity losses. Current treatments for the acromegaly patients included slow-release octreotide (Sandostatin LAR) and cabergoline. Acromegaly patients were taking from 0 to 13 medications at the time of study, depending on their comorbidities (see Table 2 for details).

### 3.3. Nonfunctioning Pituitary Adenoma Patients

Clinical characteristics of the NFA patients are included in Table 1. Like the acromegaly patients, some were taking a range of medications depending on their comorbidities as shown in Table 2.

IGF-1 levels were ascertained for all patients and a volunteer sample within the control group (33%). These are shown in Table 1. Forty-two percent of the obese acromegaly patients had high IGF-1 levels. The acromegaly patients were also categorized according to their IGF-1 status (high or controlled) and analyses carried out for all measures in the study. Patients with controlled IGF-1 had lower pain levels as measured by the DHP than those whose IGF-1 levels were high; *p* = .03.

### 3.4. Body Composition Data

BMI data collected from all participants ranged between 18.9 and 61.73, with a median of 29.46. The distribution of the various BMI across the groups can be seen in Table 1. Kruskall-Wallis analysis showed no significant differences in BMI across the three groups, *H*(2, *N* = 72) = .45, *p* = .7985. In addition, the numbers of overweight or obese participants were similar across the three groups.

### 3.5. Self-Esteem

Self-esteem responses were similar across each of the study groups, with the majority of participants reporting good self-esteem (58%); the least positive responses were recorded by 2 acromegaly and 2 control group participants. When considering only obese participants, a similar spread across the diagnostic groups was found.

### 3.6. Hospital Anxiety and Depression Scale

Using the “caseness” approach to scores on the HADS, there were 12 acromegaly patients, 7 NFA patients, and 3 control participants with anxiety or depression. However, comparison between the study groups with respect to the HADS mean scores showed no significant differences.

Examining the obese participants separately, anxiety and depression scores were worse in the patient groups; anxiety was at the 64th percentile level in acromegalic males and 61st percentile in NFPA females. Depression was at the 74th and 75th percentile for obese NFPA and acromegalic females, respectively, while in male patients NFPA men's mean score was on the 68th percentile and the acromegalic males mean score reached the 91st percentile.

### 3.7. Body Image Disturbance Questionnaire

The BIDQ asks respondents about their concern over the appearance of some parts of their body; 63% of female and 27% of male participants expressed concerns about some aspect of their body. Within each group, 54% of acromegalic women, 67% of women with NFPA, and 79% of control women expressed concerns, while 28% of acromegalic men, 22% of men with NFPA, and 43% of control men expressed concerns. A summary of the participants' expressed concerns by group appears in Table 3.

A further aspect of the BIDQ is a mean score of the seven items. Mean scores by gender and group are shown in Table 4. Analysis of patient groups showed no significant differences between either of the patient groups, or the control group for the women, or the men. A question by group examination of each quantitative response in this measure showed that the acromegaly patients expressed similar concerns and distresses on this scale as did their study counterparts. Overall a greater percentage of one or other of the two comparison groups recorded higher levels of distress or concern on every question within the BIDQ scale. However, among participants scoring 4 or more on the question as to which aspect of their body gave them concern, the NFPA and control group participants recorded a variety of bodily issues, while the acromegaly respondents were most distressed by facial changes.

### 3.8. Duke Health Profile

All participants completed the Duke Health Profile to enable comparison of QoL factors between groups. The three groups within the study were compared, and then further comparison was made with a reference group comprised of 683 primary care patients from US [31]. The 17-item measure generates health scales and disability scales, scores from which are shown in Table 5. Between-group analyses of the participants within the study found no significant differences on either the health scales or the disability scales. However when compared to the reference group of primary care patients, the acromegaly (A) and NFA patients recorded significantly lower physical health (A, *p* = .0001; NFA, *p* = .009) and perceived health (A, *p* = .0003; NFA, *p* = .04) indicating poorer health, but lower pain (A, *p* = .0001; NFA, *p* = .0001) scores indicating less pain due to health issues. However as reported above, IGF-1 levels in the acromegaly patients influenced pain scores on this measure.

When the obese participants were examined separately, the acromegaly patients' mean scores for physical health were significantly worse than the reference patients, *p* < .01, and their perceived health was also significantly worse than the reference group, *p* < .05; the NFA patients also scored lower than the reference group for perceived health, *p* < .05. There were no mean score differences on other scales for obese participant groups within the study, or when study participants were compared with the external reference group. The obese acromegaly patients' mean anxiety scores were significantly higher than the reference group, *p* < .05, indicating more dysfunction. The obese NFA patients and the obese control group participants recorded less pain than the reference group, *p* < .0001 and *p* < .01, respectively; there was no significant difference between the pain scores for the obese acromegaly patients and the US reference group.

### 3.9. Relationships between Scales

Since the study focus was body image disturbance in the three groups, the BIDQ average score and other characteristics among the study groups were examined for associations. Average BIDQ as well as BMI was related in both patient groups (acromegaly, *r* = .44, *p* < .05; NFA, *r* = .41, *p* < .05), but not in control participants. Average BIDQ was also related to HADS anxiety in all three groups (acromegaly, *r* = .57, *p* < .01; NFA, *r* = .55, *p* < .01; control, *r* = .69, *p* < .01) and to HADS depression in both patient groups (acromegaly, *r* = .68, *p* < .0001; NFA, *r* = .63, *p* < .0001).

In acromegaly patients BIDQ scores were lower when DHP physical (*r* = −.63, *p* < .01), DHP mental (*r* = −.52, *p* < .01), and DHP general health (*r* = −.54, *p* < .01) scores were higher. BIDQ scores correlated positively with anxiety (HADS and DHP *r* = .48, *p* < .05), depression (HADS *r* = .69, *p* < .0001; DHP *r* = .61, *p* < .01), and DHP pain (*r* = .46, *p* < .05) scores.

Among the NFA patients DHP mental health (*r* = −.38, *p* < .05) and DHP esteem (*r* = −.61, *p* < .01) were related to BIDQ scores; and for control participants, DHP physical (*r* = −.622, *p* < .05) and DHP pain (*r* = .57, *p* < .05) scales were correlated with BIDQ scores.

Because obesity appeared to significantly influence a number of the factors investigated in these groups, data from the obese participants (*n* = 12) were examined separately. Examination of the obese participants on a group by group basis showed that in the acromegaly patients body image problems were associated with age (*r* = .58, *p* < .05) and HADS depression (*r* = .59, *p* < .05).

Obese NFA patients' BIDQ scores were related to HADS anxiety (*r* = .62, *p* < .01) and HADS depression (*r* = .69, *p* < .01) and higher where DHP mental health (*r* = −.48, *p* < .05), DHP perceived health (*r* = −.56, *p* < .05), and DHP esteem (*r* = −.69, *p* < .01) were low. Age and self-esteem were also positively linked (*r* = .53, *p* < .05), and HADS anxiety scores were correlated with DHP perceived health (*r* = −.51, *p* < .05) and DHP pain (*r* = .48, *p* < .05) scores in this group (*n* = 18).

In the obese control participants (*n* = 7) there were no significant associations with the BIDQ average scores. HADS anxiety was associated with age (*r* = .77, *p* < .05), the DHP social scale (*r* = −.78, *p* < .05), and DHP pain (*r* = .78, *p* < .05).

## 4. Discussion

This novel study examined body image disturbance in a sample comprising three groups, two patient groups with conditions that affect their endocrine systems, and a control group of friends and relatives of the patients. The first hypothesis tested here was that body image disturbance would be more likely in acromegaly patients because of the disfiguring nature of the condition. However this was not supported by the data, with no significant differences being found between groups with respect to body image disturbance.

The second question as to how obesity affects quality of life, self-esteem, health profile, body image disturbance, and affect in these contexts was underpinned by the fact that almost 40% of study participants were obese (BMI > 29). In our study, the obese acromegaly patients reported poorer overall physical health and poorer perceived health than comparison reference groups. They also recorded higher levels of body image disturbance than their nonobese counterparts. The NFA patients were similarly affected when obese, with anxiety, depression, and perceived health concerns being associated with body image disturbance, which accords with the associations noted in the development sample [22]. Our findings suggest that overweight and obesity contribute more to reduction in quality of life and the potential for body image concerns, than the acromegaly diagnosis. This may not have been so clear had there been more significant differences between the two study groups in other respects.

The understanding that overweight and obesity relate strongly to quality of life links with a recent study of exercise, quality of life, and acromegaly that investigated whether participants reported QoL after a period of regular exercise [14]. While there were only 11 completers in that study, and the construct of body image was measured differently, those who did exercise regularly experienced a positive impact of the exercise on their self-assessed body perception. However the exercise regime did not improve the participants' mood, or their quality of life [14].

Most participants in our study, whether overweight, obese, or otherwise, regarded themselves as having high self-esteem, with responses in this area being similar across the three groups. This may be part of the reason for lack of differentiation between patient groups with respect to other study parameters; persons with high levels of self-esteem are less likely to experience body image disturbances regardless of other health diagnoses [26–28].

Concerning other aspects of mental health, while patient groups recorded more depression, the differences between groups were not statistically significant. Across the range of quality of life issues assessed by the DHP, group differences were not significant again, although both patient groups experienced more physical problems and perceived health issues than the reference sample of US primary practice patients. Pain as recorded by the DHP was worse in all three groups compared to the US patients. However it seems that combinations of these characteristics potentially contribute to differences in outcome; interactions between the scales suggested that body image disturbance was more likely when patients were obese, and when anxiety, depression, and pain were present. The examination of correlations by group demonstrated age and depression are associated with body image disturbance in acromegaly, and anxiety and depression are related to body image issues in the NFPA group; however, no such relationships were demonstrated in the control participants. This suggests that there are complex interactions among the factors we investigated that require further investigation.

Another endocrine related study recently published examined depression, pain, health, and body satisfaction in patients with Cushing's Disease, compared with controls, but found no association between their groups with respect to depression, despite general health, pain, and physical function differences [20]. However the authors also discussed the equivocal nature of the published evidence relating to depression and Cushing's Disease and pointed out that adequate treatment of the condition does not always resolve these difficulties. These findings resonate with the equivocal nature of findings in other acromegaly studies and the current study.

*Limitations*. This cross-sectional study within a relatively small sample demonstrated no significant differences among the three studied groups across these measures. This raises a question of the size of the study, and while this is almost as large as any prior study, the lack of clarity in our findings suggests a larger sample from a multicentre study may better be able to determine the scale of potential differences between these groups.

However, the lack of significant differences may be simply explained by the fact that having a diagnosis of a condition that can cause disfigurement does not necessarily mean a person experiences that effect of the condition, or further, even if the disfigurement is present, the person may not be concerned by it. This accords with findings among obese people, not all of whom experience psychological difficulty associated with their obesity [27]. Additionally, these findings agree with the study reporting adiposity in treated acromegaly patients [15]. One factor identified in the obese group is the age of onset of their obesity; people who were obese as children more often reported greater body dissatisfaction when older, than those whose obesity onset was later in life. The average participant in the current study was aged in their fifties and this may have some explanatory value for our findings; perhaps as the more general body image literature suggests, younger people would be more concerned about body image [26, 28]. On the other hand, the condition is gradual in onset, and people may more readily adjust expectations when changes are gradual in contrast to situations such as surgery for breast cancer where body image is known to be a problem for patients particularly proximal to surgery [32].

## 5. Conclusions

While the hypothesis that acromegaly might be associated with body image disturbance was not borne out, we have shown that obesity together with acromegaly can be associated with body image issues. Equally obesity and NFA may also be related to body image disturbance. Both of these findings suggest that a patient's BMI rather than their primary diagnosis will be more indicative of the likelihood that they might have body image problems. This understanding suggests the need for targeted interventions for those with either acromegaly or nonfunctioning pituitary disease when obesity is also a presenting factor. Given the equivocal nature of research regarding endocrine conditions and quality of life, further research should seek an understanding of the benefits of such targeted interventions in terms of increased quality of life, decreased body image disturbance, and overall health benefits.

## Figures and Tables

**Table 1:** Participant demographics and characteristics.

	Acromegaly *N* = 32	Nonfunctioning pituitary adenoma *N* = 29	Control *N* = 21
Age			
Mean ± SD	55.3 ± 15.3	62.5 ± 15.0^∗^	50.3 ± 14.4^∗^
Range	25–83	26–91	25–70
Median	58	65	53
Gender			
Female	13	6	14
Male	19	23	7
Ethnicity			
NZ European	27	23	15
Maori/PI	4	6	5
Other	1	0	1
Relationship status^∗^			
Partnered	26	19	14
Single	6	10	6
*Clinical characteristics *			
BMI^∗^			
Mean ± SD	30.89 ± 6.88	30.7 ± 6.43	29.4 ± 5.16
Range	23.1–61.7	18.9–44.4	22.1–38.8
Obese	*N* = 16 [50%]	*N* = 19 [66%]	*N* = 7 [44%]
IGF-1 levels^#^			
Low	*N* = 3	*N* = 10	—
Normal	*N* = 15	*N* = 19	*N* = 7
High	*N* = 13	—	—
Initial surgery^∗^ (1977–2012)			
TSH, transfrontal or craniotomy	*N* = 24	*N* = 22	—
Pending	*N* = 3	—	—
Second surgery^∗^ (1978–2012)			
TSH	4	3	—
Pending	1	—	—
Radiotherapy^∗^	10	10	—
Number of current medications			
0-1	10	6	13
2-3	8	12	4
>3	14	11	4

Notes: ^∗^missing data; ^#^low or high IGF-1 levels were defined as those outside the age and sex matched reference range.

**Table 2:** Participant comorbidities.

	Acromegaly	NFA	Control
Diabetes	4	3	2
Impaired glucose tolerance	3	0	1
Heart disease	3	7	1
High cholesterol	16	8	4
Thyroid problems	10	10	1
Goitre	2	1	0
Thyroidectomy	2	2	1
Visual loss	7	12	7
Ceased driving, visual loss	4	2	1
Bowel polyps	10	4	2
Bowel cancer	3	0	0
Stroke	2	2	3
TIA	3	1	3
High blood pressure	14	11	8
Carpal tunnel syndrome	11	2	5
Arthritis	15	13	2
Joint replacement	3	3	2
Obstructive sleep apnoea	10	3	0
Prescribed CPAP	4	1	0
Cancer	9	6	0
Types of cancer reported:	Squamous, prostate, breast, colon, skin	Prostate, brain	—
Asthma	0	3	3

**Table:** (a) All participants expressing concerns (*n* = 37)

	Women's concerns (*n* = 22)		Men's concerns (*n* = 15)	
All groups	*N*	Concerns	*N*	Concerns
	22		15	
Acromegaly	7	Being obese or weight gain (3), facial changes (2), hands too large (2), xanthelasma (1)	7	Jaw/facial features (5), body size (2), hands and feet (3)
NFPA	4	Weight (2), “fat parts” (1), stretch marks (1)	5	Weight (3), size of stomach (1), loss of muscle (1)
Control	11	Weight (5), fat stomach/body (3), hairiness (3), large hands, jaw (1), teeth (1), wrinkles/old age (1)	3	Nose (1), stomach (1), expanding waist/weight (1)

**Table:** (b) Obese participants expressing concerns (*n* = 17)

	Women's concerns (*n* = 9)		Men's concerns (*n* = 8)	
All groups	*N*	Concerns	*N*	Concerns
	9		8	
Acromegaly	3	Being obese or weight gain (3)	3	Jaw/facial features (2), body size (2)
NFPA	2	Weight (2), “fat parts” (1)	3	Weight (2), size of stomach (1)
Control	4	Weight (2), fat stomach/body (3), hairiness (3)	2	Nose (1), expanding waist/weight (1)

**Table 4:** Mean scores on the BIDQ by group.

	Women		Men	
	*N*	Mean (SD)	*N*	Mean (SD)
All participants				
Acromegaly	13	1.67 (0.89)	19	1.61 (0.70)
NFPA	6	1.83 (0.77)	23	1.47 (0.73)
Control	14	1.69 (0.84)	7	1.67 (1.24)
Obese participants				
Acromegaly	4	1.88 (0.66)	12	1.95 (0.56)
NFPA	3	2.05 (0.46)	15	1.56 (0.70)
Control	4	1.79 (0.50)	3	2.43 (1.27)

**Table 5:** Health and Disability Scales of the Duke Health Profile by participant group.

	Acromegaly *N* = 32	NFP tumor *N* = 29	Control *N* = 21	Reference patients *N* = 683
	Mean ± SD	Mean ± SD	Mean ± SD	Mean ± SD
Health scales				
Physical	51.25^a^ ± 26.85	56.07^b^ ± 24.39^∗^	64.76 ± 24.82	67.1^ab^ ± 22.2
Mental health	76.61 ± 21.96	72.41 ± 19.58	81.00 ± 18.04	72.8 ± 20.6
Social health	64.69 ± 23.96	61.85 ± 20.0^∗^	68.57 ± 18.78	67.6 ± 17.6
General health	64.36 ± 18.05	63.09^c^ ± 16.74	71.33 ± 17.42	69.2^c^ ± 15.4
Perceived health	59.38^d^ ± 36.89	68.97 ± 36.39	69.05 ± 33.45	76.9^d^ ± 26.4
Self-esteem	72.50 ± 23.28	73.10 ± 21.56	72.86 ± 18.21	76.9 ± 18.3

Disability scales				
Anxiety	33.33 ± 20.52	34.20 ± 17.59	25.00 ± 17.87	27.9 ± 18.3
Depression	32.50 ± 21.40	37.59 ± 18.26	25.24 ± 20.15	30.8 ± 21.4
Pain	53.13^e^ ± 35.78	46.55^f^ ± 32.54	42.86^g^ ± 32.75	67.6^efg^ ± 17.6
Disability	18.75 ± 35.36	17.24 ± 33.48	4.76 ± 15.04	16.5 ± 28.3

Note: ^∗^missing data. Superscripts denote pairs that differ significantly, *p* < .05.
